# How useful is the machine perfusion in liver transplantation? An answer from a national survey

**DOI:** 10.3389/fsurg.2022.975150

**Published:** 2022-09-22

**Authors:** Irene Scalera, R. De Carlis, D. Patrono, E. Gringeri, T. Olivieri, D. Pagano, Q. Lai, M. Rossi, S. Gruttadauria, F. Di Benedetto, U. Cillo, R. Romagnoli, L. G. Lupo, L. De Carlis

**Affiliations:** ^1^ Hepatobiliary and Liver Transplant Unit, Department of Emergency and Organ Transplantation, University Hospital Policlinic of Bari, Bari, Italy; ^2^ Department of General Surgery and Transplantation, ASST Grande Ospedale Metropolitano Niguarda, Milan, Italy; ^3^ General Surgery 2U-Liver Transplant Centre, A.O.U. “Città della Salute e della Scienza”, Turin, Italy; ^4^ Hepatobiliary Surgery and Liver Transplantation Unit, University Hospital of Padua, Padua, Italy; ^5^ Hepato-Pancreato-Biliary Surgery and Liver Transplant Center, University of Modena and Reggio Emilia, Modena, Italy; ^6^ Department for the Treatment and the Study of Abdominal Diseases and Abdominal Transplantation, IRCCS-ISMETT, UPMC, Palermo, Italy; ^7^ Department of Surgery and Medical and Surgical Specialties, University of Catania, Catania, Italy; ^8^ Liver Transplant Unit, Sapienza University of Rome, Rome, Italy; ^9^ Department of Medicine and Surgery, University of Milano-Bicocca, Milan, Italy

**Keywords:** liver donor, machine perfusion, steatosis, early allograft dysfunction, liver transplantation

## Abstract

Machine perfusion (MP) has been shown worldwide to offer many advantages in liver transplantation, but it still has some gray areas. The purpose of the study is to evaluate the donor risk factors of grafts, perfused with any MP, that might predict an ineffective MP setting and those would trigger post-transplant early allograft dysfunction (EAD). Data from donors of all MP-perfused grafts at six liver transplant centers have been analyzed, whether implanted or discarded after perfusion. The first endpoint was the negative events after perfusion (NegE), which is the number of grafts discarded plus those that were implanted but lost after the transplant. A risk factor analysis for NegE was performed and marginal grafts for MP were identified. Finally, the risk of EAD was analyzed, considering only implanted grafts. From 2015 to September 2019, 158 grafts were perfused with MP: 151 grafts were implanted and 7 were discarded after the MP phase because they did not reach viability criteria. Of 151, 15 grafts were lost after transplant, so the NegE group consisted of 22 donors. In univariate analysis, the donor risk index >1.7, the presence of hypertension in the medical history, static cold ischemia time, and the moderate or severe macrovesicular steatosis were the significant factors for NegE. Multivariate analysis confirmed that macrosteatosis >30% was an independent risk factor for NegE (odd ratio 5.643, *p* = 0.023, 95% confidence interval, 1.27–24.98). Of 151 transplanted patients, 34% experienced EAD and had worse 1- and 3-year-survival, compared with those who did not face EAD (NoEAD), 96% and 96% for EAD vs. 89% and 71% for NoEAD, respectively (*p* = 0.03). None of the donor/graft characteristics was associated with EAD even if the graft was moderately steatotic or fibrotic or from an aged donor. For the first time, this study shows that macrovesicular steatosis >30% might be a warning factor involved in the risk of graft loss or a cause of graft discard after the MP treatment. On the other hand, the MP seems to be useful in reducing the donor and graft weight in the development of EAD.

## Introduction

Extended criteria grafts are increasingly used worldwide (1, 2) to fight the persistent shortage of organs for liver transplantation (LT). However, implantation of these grafts is challenging, being linked to more intraoperative complications, such as post reperfusion syndrome (3, 4), or post-transplant problems like early allograft dysfunction (EAD) (5), renal injury (6), or biliary complications (7). All these complications can cost graft loss or patient's life (8). The duration of static cold ischemia time (S-CIT) plays a significant role in this context, as nonstandard grafts do not tolerate long periods of storage on ice (9). Indeed, S-CIT is a component of several donor risk formulas, such as the donor risk index (DRI) (10), the EuroTransplant donor risk index (11), the Survival Outcomes Following Liver Transplantation (SOFT) score (12), and the Balance of Risk score (13). The pathophysiology of S-CIT is triggered by the lack of oxygen supply during the cold ischemic phase. Cellular energetic processes are slower and many catabolites accumulate to be released in the recipient blood (14). The clinical impact of this process may manifest after the graft implantation with post reperfusion syndrome or even compromise the patient's survival after transplantation (15, 16). In addition, during S-CIT, there is no opportunity to assess graft viability and the transplant surgeon may be discouraged to use extended criteria grafts.

In recent years, the liver transplant community has focused on the administration of oxygen at low- or body-temperature using machine perfusions (MP) *ex vivo*. The results are positive even with nonstandard grafts (17). The first published study with long-term follow-up showed that hypothermic MP (HMP) treatment of deceased-cardiac donors’ donation after circulatory death (DCD) livers protect grafts and significantly reduce ischemic cholangiopathy and graft loss rates (18). In Italy, since 2015, several liver transplant centers have started to use the MP and its benefits have marked a positive trend to use. Therefore, these devices have slowly ushered in a promising new era for LT.

However, the MP is not immune to drawbacks: costs are still high for the most widely used modalities, namely HMP and normothermic MP (NMP). In Italy, a disposable kit for perfusing a graft costs about 8,000–12,000 euros for the Liver Assist Machine (Organ Assist, Netherlands). The NMP needs packed red cells, drugs, and nutritional solution (17). All devices need qualified personnel on call as well. In addition, the primary nonfunction (PNF) rate for all MPs is low, ranging 1.2%–4%, but is still reported. The frequency of EAD is 10% after NMP treatment and 33% after HMP (9). All these complications increase the overall cost of MP. In addition, not all machine-perfused grafts fulfill viability test criteria to be used and they are obviously discarded after MP treatment. The discard rate for NMP reported in a randomized controlled trial is 12% (9), but there are no data on the risk factors of donor discard with MP. If in the MP era, marginal grafts are used more frequently and successfully, then for MP preserved grafts, the label “marginal” needs to be redefined to understand how far we can push the donor risk boundaries today. Finally, more marginal grafts have been used (1, 2) but there is no clear information on the EAD risk for these grafts after having been oxygenated in MP. Therefore, the aim of this study is to investigate the utility of MP and to delineate the pretransplant risk factors for setting up unnecessary MP.

## Materials and methods

### Study design

Data of MP grafts, either by HMP or NMP, were collected from six Italian liver transplant centers. The study period was from the first Italian case in 2015 up to September 2019. Both donation after brain death (DBD) and donation after circulatory death (DCD) were considered.

**Figure 1:**
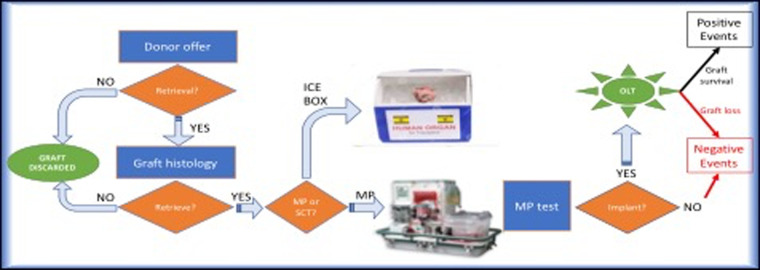
Decision-making process from the donor offer to the transplant and graphic presentation of the two study groups.

The first endpoint was to identify the risk factors predicting negative events (NegE). NegE were defined as all perfused but discarded livers and implanted grafts lost after LT. Positive events (PosE) included all successfully transplanted perfused grafts (Figure 1).

Extended criteria grafts were arbitrarily defined as grafts from donors with at least two of the worst interquartile of pretransplant characteristics if the graft was from DBD, or one of them if from DCD. This way was done to better identify the “very extended” criteria in our specific donor population and not considering “extended” criteria from the literature. MP was used mainly for grafts with some very warning characteristics that could rarely be reported in the literature. Therefore, published definitions of marginal grafts would not have fit for this study of “very marginal” donors. This group of grafts was, therefore, analyzed if significant for NegE with the aim of defining a new concept of “marginal graft” in the MP era.

The second endpoint was to analyze the role of MP in terms of post-transplant EAD rates. First, the impact of EAD on patient and graft survival was studied. Finally, donor- and graft-related risk factors of EAD were investigated.

National guidelines for liver transplantation were used for organ donation eligibility and graft allocation. All surgeries were performed using a piggyback technique for caval reconstruction.

### Definitions of variables

Histology features were reported according to the grade of macro- and microvesicular steatosis and greater or less than 30%. Ishak fibrosis was cited if greater than grade 1.

The donor risk index was calculated according to the formula published by Feng et al. (10). EAD was defined if the case met Olthoff's criteria (19). PNF was considered if death or retransplantation due to liver failure in the first postoperative week and if the cause was not related to acute rejection or vascular complications (20). Finally, S-CIT was the elapsed time between donor cross-clamp and MP initiation.

### Modalities of MP and NRP

For DBD grafts, every center followed its own policy in deciding whether to store the graft in the ice box until the implantation (S-CIT) or to use the MP. For MP grafts, the device was set up at the transplant center after a variable period of S-CIT. All of the grafts were oxygenated *ex situ*, most in hypothermic mode and a few of them in normothermic setting, for at least 2 h until the recipient hepatectomy was completed. For both modalities, the vessel perfusion was used at manually controlled pressure and flow. The arterial circuit was set up at 40 mmHg with pulsatile perfusion pressure, 60 beats for minute with 1,000 ml/min of flow. Portal flow was set up at 6 mmHg continue perfusion pressure with flow up to 2 L/min. University of Wisconsin MP fluid was used for HMP. The grafts were flushed with saline solution or Celsior fluid and implanted after the HMP treatment.

For DCD grafts, all cases were treated with normothermic regional perfusion (NRP) followed by *ex vivo* MP. Only grafts with macrosteatosis ≤30% and Ishak grade ≤1 at biopsy were considered available. National criteria for proceeding with liver retrieval on NRP were ALT <1,000 UI/L (or negative transaminase trend) and negative lactate trend (21).

### Statistical analysis

Donor and graft characteristics are listed in Table 1. Quantitative variables are described in medians and ranges. Qualitative variables are reported in total numbers and percentages.

**Table 1:** Univariate analysis of donor characteristics for the negative events.

Variables	Positive events 136 cases *n* (%)	Negative events 22 cases *n* (%)	*p*-value
Age, years, median (range)	63 (13–96)	62 (49–83)	0.80
Interquartile			
<55	36 (26)	5 (23)	0.50
55–63	32 (24)	8 (36)	
64–75	34 (25)	6 (27)	
>75	34 (25)	3 (14)	
BMI, median (range)	26 (16–62)	26 (22–35)	0.55
Interquartile			
<25	52 (38)	10 (46)	0.66
25–26	9 (7)	2 (9)	
27–29	42 (31)	4 (18)	
>29	33 (24)	6 (27)	
ICU, days median (range)	3 (0–20)	2 (0–24)	0.93
Interquartile			
<2	53 (39)	12 (55)	0.57
2–3	17 (13)	1 (5)	
4–6	37 (27)	5 (23)	
>6	29 (21)	4 (18)	
Cause of death			
HBI	24 (18)	5 (24)	0.07
Trauma	15 (11)	1 (5)	
CVA	75 (55)	7 (33)	
Other	22 (16)	8 (38)	
Gender, male, *n* (%)	84 (62)	13 (63)	>0.99
Donor DCD	43 (32)	11 (50)	0.14
DRI, median (range)	1.91 (1.6–4.1)	1.98 (1.6–2.5)	**0.01**
Interquartile			
<1.79	45 (33)	3 (14)	**0.01**
1.79–1.91	40 (30)	4 (18)	
1.92–2.09	16 (12)	9 (41)	
>2.09	35 (25)	6 (27)	
Sodium, mEq/L, median (range)	148 (114–182)	147 (140–166)	0.71
Interquartile			
<143	42 (31)	8 (36)	0.52
1.44–148	31 (23)	7 (32)	
149–154	27 (27)	3 (14)	
>154	26 (19)	4 (18)	
GGT, UI/L, median (range)	34 (7–430)	53 (18–147)	>0.99
Interquartile			
<20	72 (53)	14 (63)	0.83
20–36	21 (16)	3 (14)	
37–76	22 (16)	2 (9)	
>76	21 (15)	3 (14)	
Bilirubin, mg/dl, median (range)	0.6 (0.1–4.4)	0.8 (0.2–5)	0.91
Interquartile			
<0.34	85 (63)	10 (45)	0.20
0.34–0.60	19 (14)	3 (14)	
0.61–0.98	17 (12)	3 (14)	
>0.98	15 (11)	6 (27)	
ALT, UI/L, median (range)	40 (6–1,803)	68 (7–505)	0.23
Interquartile			
<22	86 (63)	8 (36)	0.07
22–49	17 (13)	4 (18)	
50–103	16 (12)	6 (27)	
>103	17 (12)	4 (18)	
AST, UI/L, median (range)	54 (9–1,782)	102 (8–465)	0.25
Interquartile			
<27	90 (66)	10 (45)	0.10
28–55	18 (13)	3 (14)	
56–133	15 (11)	3 (14)	
>133	13 (10)	6 (27)	
Diabetes^a^	18 (13)	0	0.14
Hypertension^b^	36 (26)	12 (54)	**0.04**
Dyslipidemia^c^	14 (10)	2 (9)	0.41
Inotrope^d^	81 (65)	8 (47)	0.18
Anti-HBc positive^e^	20 (16)	3 (18)	0.73
CMV IgG positive^f^	74 (69)	5 (39)	0.06

^a^ Missing information for 36 donors.

^b^ Missing information for 71 donors.

^c^ Missing information for 77 donors.

^d^ Missing information for 18 donors.

^e^ Missing information for 39 donors.

^f^ Missing information for14 donors.

BMI, body mass index; ICU, intensive care unit; DRI, donor risk index; HBI, hypoxic brain injury; CVA, cardiovascular accident.

Bold values are statistically significant.

The Kolmogorov–Smirnov test was performed to evaluate the normal distribution of continuous variables. Continuous variables with parametric distribution were compared with the nonpaired sample Student’s *t* test, and variables with skewed distribution with the Mann–Whitney test. Qualitative variables were analyzed with the chi-square or Fischer test, as appropriate. Multivariable logistic regression analysis was performed considering only variables significant to univariate analysis. Odds ratio (OR) and 95% confidence intervals (95% CI) were reported. The Kaplan–Meier modality was used for survival curves, and the log-rank test was used for survival comparison. Results were considered significant if the *p*-value was <0.05. IBM® SPSS (version 24) was used for statistical analyses

## Results

From 2015 to September 2019, 158 grafts were retrieved and preserved with an MP. A total of 151 livers were implanted; therefore, seven grafts were not used, with a discard rate of only 4.4% after MP treatment. After transplant, 15/151 (9.9%) patients lost their grafts (with five cases for PNF, three for vascular complications, three for sepsis, and four for patient-related causes). Therefore, the NegE group consisted of 22/158 (13.9%) grafts. The PosE group consisted of 136/158 (86.1%) patients.

DCD grafts were 53 from Maastricht 3 (33.54%) and 1 from Maastricht 2. Most of the livers were perfused with HMP (*n* = 144, 91.1%).

**Figure 2:**
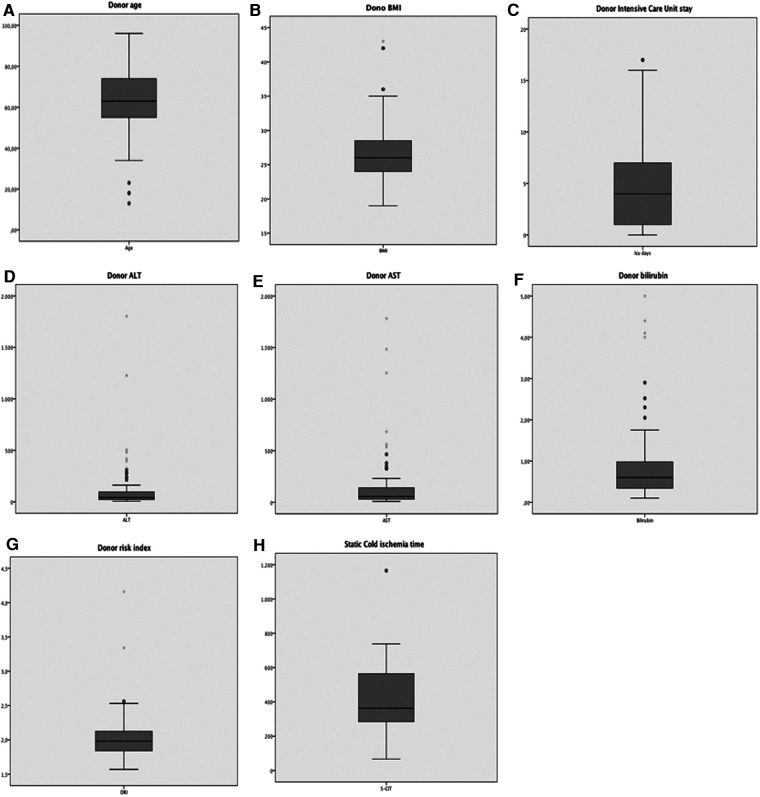
Graphic display of the distribution of the main donor parameters of all 158 donors included in the study. (**A**) Age; (**B**) BMI; (**C**) Intensive Care Unit stay; (**D**) ALT; (**E**) AST; (**F**) bilirubin; (**G**) donor risk index; (**H**) S-CIT duration.

Figure 2 graphically illustrates the donor characteristics of all the 158 grafts. The median donor age was 63 years (range = 13–96) and 69 (44%) donors were older than 65 years. The median body mass index (BMI) was 26 (range = 16–62) and 22% of donors were obese. Notably, 9 (5.7%) donors belonged to obesity class III.

The median stay in the intensive care unit (ICU) was 3 days (range = 1–24). Forty-seven (29.7%) donors had deranged transaminases at the time of procurement. The median donor ALT peak value was 49 IU/L (range = 6–1,803), and the median AST peak value was 55 IU/L (range = 8–1,782). Fourteen (8.9%) donors had a peak value of total bilirubin >1.2 mg/dl at the time of procurement, with a median value observed in the whole population of 0.6 mg/dl (range = 0.1–5.0). One hundred forty-three (90.5%) donors had a DRI >1.7 (median 1.9; range = 1.6–4.2). S-CIT lasted longer than 7 h in 41 cases (25.9%). Notably, one graft had up to 20 h of storage in ice before MP (Figure 2).

Table 1 describes the comparison between the NegE and PosE groups in terms of donor characteristics. Donor age, BMI, cause of death, sex, transaminases, GGT, bilirubin, and sodium level were similar in both groups. The median DRI was significantly higher in NegE than PosE (2.0 vs. 1.9; *p* = 0.01), and the groups had different distribution among the interquartiles. When donor medical history was available, the rates of diabetes, dyslipidemia, cytomegalovirus IgG, and anti-HBc positivity were not statistically different between the groups. There were significantly more hypertensive donors in NegE (Table 1).

A different proportion of MP type was noted: in PosE, 93% of grafts were perfused by HMP, compared with 77% in NegE (*p* = 0.03). Median S-CIT was similar in both groups, with a different interquartile distribution (*p* = 0.01) (Table 2). Biopsy results at the time of retrieval showed similar rates of microvesicular steatosis grade and Ishak score of fibrosis. NegE cases had more grafts with moderate-to-severe macrovesicular steatosis (32% vs. 7%, *p* = 0.02) (Table 2).

**Table 2:** Univariate analysis of graft histology features, type of machine perfusion adopted, and S-CIT for the negative events.

Variables	Positive events 136 cases *n* (%)	Negative events 22 cases *n* (%)	*p*-value
Type of machine			
HMP:NMP	127:9	17:5	**0.03**
%HMP	93%	77%	
S-CIT, hours, median (range)	6 (2–19)	4 (1–6)	0.14
Interquartile			
<4	15 (11)	9 (41)	**0.01**
4–7	83 (61)	10 (46)	
7–8	11 (8)	1 (4)	
>8	27 (20)	2 (9)	
Macrosteatosis >30%	9 (7)	7 (32)	**0.02**
Microsteatosis >30%	23 (17)	3 (14)	>0.99
Fibrosis Ishak grade >1	10 (7)	1 (5)	>0.99

S-CIT, static cold ischemia time; HMP, hypothermic machine perfusion; NMP, normothermic machine perfusion.

Bold values are statistically significant.

All significant factors at univariate analysis were considered to build a multivariable logistic regression model for the risk of NegE. After introducing five different variables in the model (DRI, presence of hypertension, type of MP, duration of S-CIT expressed in interquartiles, and grade of macrovesicular steatosis), only macrosteatosis >30% was found to be a significant variable, with an odds ratio of 5.643 (95% CI, 1.27–24.98; *p* = 0.023) (Table 3).

**Table 3:** Multivariate analysis of the donor and grafts risk factors predicting the negative events.

Variables	*p*-value	Odds ratio	95% CI
Hypertension	0.13	0.65	0.13–3.28
Type of machine (HMP)	0.82	0.79	0.10–6.16
DRI	0.15	3.98	0.62–25.66
S-CIT, hours	0.20	0.42	0.09–1.95
Macrosteatosis >30%	**0.023**	**5.64**	**1.27–24.98**

S-CIT, static cold ischemia time; HMP, hypothermic machine perfusion; DRI, donor risk index; CI, confidence interval.

Bold values are statistically significant.

Separate analysis was performed for the two subgroups of NegE. Table 4 shows a comparison of implanted and survived cases vs. discarded grafts and survived vs. lost grafts.

**Table 4:** Subgroups analysis of the donor and grafts risk factors predicting the discarded grafts and the grafts lost.

Variables	Implanted 136 cases *n* (%)	Not implanted 7 cases *n* (%)	*p*-value	Implanted 136 cases *n* (%)	Grafts lost 15 cases *n* (%)	*p*-value
Age, years median	63	62	0.73	63	61	0.72
BMI median	26	27	0.76	26	25	0.43
ICU, days median	3	1	0.42	3	5	0.96
Cause of death						
HBI	24 (18)	2 (33)	0.07	24 (18)	3 (20)	0.39
Trauma	15 (119	0 (0)		15 (119	1 (7)	
CVA	75 (55)	1 (17)		75 (55)	6 (40)	
Other	22 (16)	3 (50)		22 (16)	5 (33)	
Gender male *n* (%)	84 (62)	4 (50)	0.68	84 (62)	10 (67)	0.79
Donor DCD	43 (32)	3 (43)	0.68	43 (32)	8 (53)	0.15
DRI median	1.91	1.98	0.03	1.91	1.98	0.16
Sodium mEq/L, median	148	148	0.96	148	147	0.84
GGT UI/L, median	34	97	0.24	34	29	0.69
Bilirubin, mg/dl, median	0.60	2.30	0.32	0.60	2.30	0.90
ALT, UI/L, median	40	240	0.60	40	58	0.31
AST, UI/L, median	54	177	0.05	54	55	>1
Type of machine						
HMP:NMP	127:9	5:2	0.09	127:9	12:3	0.37
%HMP	93%	71%		93%	80%	
S-CIT, hours median	6	3	0.36	6	5	0.41
Macrosteatosis >30%	7(5)	4 (57)	**0.01**	7(5)	3 (20)	0.07

BMI, body mass index; ICU, intensive care unit; DRI, donor risk index; S-CIT, static cold ischemia time; HMP, hypothermic machine perfusion; NMP, normothermic machine perfusion; HBI, hypoxic brain injury; CVA, cardiovascular accident.

Bold values are statistically significant.

### Marginal donors

Extended criteria donors (ECDs) were defined as all donors with at least of two of the following characteristics: DCD, age > 65 years, BMI > 30, ICU stay >6 days, DRI > 1.9, ALT > 104 UI, AST > 134 UI, bilirubin > 1.2 mg/dl, S-CIT longer than 8 h, and macrosteatosis > 30% (Table 5). Of the 54 so-called marginal donors, 27% had a negative event, compared with the remaining (9%, *p*-value of 0.01) defined as standard criteria donors (SCDs).

**Table 5:** List of donor parameters: occurrence of two or more of these factors was considered to define a marginal donor for grafts treated by MP.

Donor variables
DCD
Age > 65 years
BMI > 30
ICU stay > 6 days
DRI > 1.9
ALT > 104 UI/L
AST > 134 UI/L
Bilirubin > 1.2 mg/dl
S-CIT > 8 h
Macrosteatosis > 30%

BMI, body mass index; ICU, intensive care unit; DRI, donor risk index; S-CIT, static cold ischemia time.

### Analysis of cases of early allograft dysfunction

**Figure 3:**
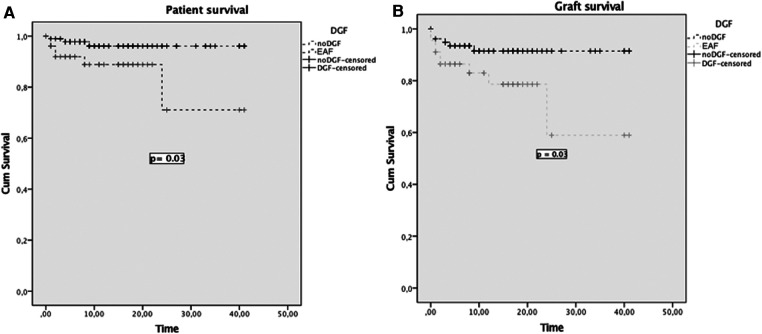
Patient (**A**) and graft survival (**B**) curves for patients who developed EAD and patients who did not (NoEAD).

For the analysis of EAD, 151 donors of MP stored and implanted grafts were considered and the rate was 34% (52 patients vs. 99 no EAD). Notably, EAD had a significant impact on patient survival at 1 and 3 years post liver transplant: 96% and 96% for patients who did not develop an EAD (noEAD) vs. 89% and 91% for EAD patients (*p* = 0.03), respectively. A similar difference was noted between grafts survival curves: at 1 and 3 years 93% and 91% for noEAD and 86% and 79% for EAD patients (*p* = 0.03) (Figure 3).

Table 6 summarizes all donor characteristics. In both groups, EAD and noEAD, nearly one-third of grafts were from DCD donors, and more than two-third had DRI > 1.7. Laboratory results, median donor age, BMI, and diseases mentioned in medical history were similar in both groups. The type of MP used, duration of S-CIT, and histology features were also comparable (Table 7).

**Table 6:** Univariate analysis of donor factors for developing an EAD.

Variables	No EAD 99 patients *n* (%)	EAD 52 patients *n* (%)	*p*-value
Age, years median (range)	66 (35–87)	63 (47–72)	0.43
Interquartile			
<55	28 (29)	11 (21)	**0.01**
55–63	21(21)	17 (33)	
64–75	20 (20)	18 (35)	
>75	30 (30)	6 (11)	
BMI median (range)	25 (21–43)	26 (23–42)	0.88
Interquartile			
<25	40 (41)	19 (36)	0.89
25–26	8 (8)	3 (6)	
27–29	27 (27)	17 (33)	
>29	24 (24)	13 (25)	
ICU, days, median (range)	3 (0–17)	5 (1–16)	0.90
Interquartile			
<2	42 (42)	18 (35)	0.09
2–3	9 (10)	8 (15)	
4–6	31 (31)	10 (19)	
>6	17 (17)	16 (31)	
Cause of death			
HBI	19 (19)	8 (15)	0.21
Trauma	12 (12)	4 (8)	
CVA	47 (48)	34 (65)	
Other	21 (21)	6 (12)	
Gender male, *n* (%)	60 (61)	34 (65)	0.6
Donor DCD	33 (33)	18 (34)	>0.99
DRI median (range)	1.91 (1.57–2.54)	1.91 (1.57–4.16)	0.68
Interquartile			
<1.79	32 (33)	16 (31)	0.75
1.79–1.91	26 (26)	17 (33)	
1.92–2.09	16 (15)	5 (9)	
>2.09	26 (26)	14 (27)	
Sodium, mEq/L, median (range)	148 (135–170)	153 (138–182)	0.09
Interquartile			
<143	32 (33)	15 (29)	0.07
1.44–148	27 (27)	9 (17)	
149–154	27 (27)	12 (23)	
>154	13 (13)	16 (31)	
GGT, UI/L, median (range)	22 (11–324)	56 (11–148)	0.87
Interquartile			
<20	52 (53)	30 (58)	0.95
20–36	16 (16)	8 (15)	
37–76	16 (16)	7 (14)	
>76	15 (15)	7 (13)	
Bilirubin, mg/gl, median (range)	0.68 (0.17–2.5)	0.54 (0.3–1.75)	0.61
Interquartile			
<0.34	63 (64)	29 (56)	0.42
0.34–0.60	11 (11)	11 (21)	
0.61–0.98	14 (14)	6 (12)	
>0.98	11 (11)	6 (11)	
ALT, UI/L, median (range)	23 (6–1,803)	36 (16–418)	0.49
Interquartile			
<22	62 (63)	30 (58)	0.56
22–49	11 (11)	10 (19)	
50–103	13 (13)	7 (13)	
>103	13 (13)	5 (10)	
AST, UI/L, median (range)	32 (12–1,782)	48 (11–171)	0.81
Interquartile			
<27	67 (68)	31 (60)	0.18
28–55	10 (10)	11 (21)	
56–133	10 (10)	7 (13)	
>133	12 (12)	3 (6)	
Diabetes^a^	11 (11)	7 (13)	0.6
Hypertension^b^	24 (24)	29 (38)	0.17
Dyslipidemia^c^	10 (10)	6 (12)	0.85
Inotrope^d^	54 (58)	31 (72)	0.13
Anti-HBc positive^e^	12 (13)	10 (21)	0.22
CMV IgG positive^f^	53 (66)	26 (67)	>0.99

^a^ Missing information for 35 donors.

^b^ Missing information for 69 donors.

^c^ Missing information for 75 donors.

^d^ Missing information for 15 donors.

^e^ Missing information for 32 donors.

^f^ Missing information for 11 donors.

EAD, early allograft dysfunction; BMI, body mass index; ICU, intensive care unit; DRI, donor risk index; HBI, hypoxic brain injury; CVA, cardiovascular accident.

Bold values are statistically significant.

**Table 7:** Univariate analysis of graft histology features, type of machine perfusion used, and S-CIT for EAD.

Variables	No EAD 99 patients *n* (%)	EAD 52 patients *n* (%)	*p*-value
Type of machine			
HMP:NMP	92:7	47:5	0.75
%HMP	93%	90%	
S-CI,T hours median (range)	8 (2–12)	7 (6–19)	0.44
Interquartile			
<4	13 (13)	6 (11)	0.95
4–7	60 (61)	31 (60)	
7–8	7 (7)	5 (10)	
>8	19 (19)	10 (19)	
Macrosteatosis >30%	6 (6)	6 (12)	0.34
Microsteatosis >30%	17 (17)	9 (17)	>0.99
Fibrosis Ishak grade >1	7 (7)	4 (8)	>0.99

EAD, early allograft dysfunction; S-CIT, static cold ischemia time; HMP, hypothermic machine perfusion; NMP, normothermic machine perfusion.

Characteristics of the recipients are shown in Table 8: both groups of patients had similar age, BMI, cause of cirrhosis, hepatocellular carcinoma (HCC) rate, and MELD score at the time of transplant.

**Table 8:** Recipient characteristics for patients who developed EAD compared who did not develop (NoEAD).

Variables	No EAD 99 patients *n* (%)	EAD 52 patients *n* (%)	*p*-value
Age, years median (range)	58 (45–69)	59 (17–70)	0.88
BMI median (range)	25 (18–34)	26 (18–34)	0.35
Gender male, *n* (%)	77 (78%)	42 (81%)	0.71
Cause of cirrhosis			
Viral	52 (53)	30 (58)	0.61
ALD	31 (31)	11 (21)	0.25
NASH	19 (19)	3 (6)	0.03
Other	14 (14)	11 (21)	0.36
HCC	67 (68)	36 (69)	0.68
MELD score	11(6–26)	10 (5–39)	0.19

EAD, early allograft dysfunction; HCC, hepatocellular carcinoma; MELD, model of end stage liver disease; ALD, alcoholic liver disease; NASH, non alcoholic steatohepatitis.

## Discussion

### Negative and positive events

This is the first study exploring the possible pretransplant risk factors to discard a liver graft after MP preservation or failure after implantation to help the surgeon determine whether it is worthwhile to set up an MP.

Univariate analysis has confirmed the utility of MP as widely mentioned in the literature (9, 17, 22–24): grafts from elderly donors, or with those with very high BMI, or with deranged laboratory test can be saved by MP (Table 1). In fact, recently a trial has indeed shown that HMP definitely reduces ischemia reperfusion injury in ECD compared with grafts from SCD (17). In addition MP, used after NRP, allowed the safe use of DCD grafts, which would not have been used without this tool due to very long donor warm ischemia time (WIT) (21, 25). Because of the long standoff time (20 min), in Italy, DCD grafts have donor WIT longer than 30 min, which would be an exclusion criteria for the use of these grafts. In this study, 43 DCD grafts were successfully implanted and the percentage of DCD vs. DBD was similar in the NegE (11 vs. 11) and PosE groups (43 vs. 93, *p* = 0.14). There are no data comparing the sequential of NRP and *ex situ* preservation vs. NRP alone, but in an evidence-based position paper recently published by the Italian Society of Organ Transplantation (SITO), sequential use in DCD was recommended (26).

Interestingly, this study uncovers a gray area for the use of MP in a clinical setting. Among the significant parameters at univariate analysis, only moderate/severe macrosteatosis was an independent factor to discard the graft after MP preservation or to have graft loss after transplantation. Neither moderate or severe microsteatosis nor Ishak grade >1 limited the usefulness of MP, but macrosteatosis >30% remained a warning factor for grafts even after MP treatment (Table 3). NegE had 32% of moderate/severe steatosis vs. 7% in PosE (*p* = 0.02). Steatotic livers have been often linked to a worse post-transplant outcome, particularly post reperfusion syndrome, EAD, renal injury, and postoperative mortality (27–29). This new result may dictate greater care in dealing with steatotic grafts, especially before preserving them in an MP. Considered that donor BMI is a surrogate marker of the grade of liver steatosis and is increasing worldwide (1, 2), research needs to be addressed and implemented more in this field. Steatosis is still one the most frequent causes to discard a liver, with rates ranging 13%–28% (30); therefore, there is a tremendous interest in saving these grafts. Separate analysis with the two subgroups of NegE vs. implanted grafts showed that the rate of macrosteatosis was significant in discarded grafts (Table 4) but not in lost grafts subgroup. The numbers are very small, but these data might stress the concept that it needed to treat these very steatotic grafts in the MP to have more functional livers available. If microsteatosis does not scare any surgeon, clinical data on *ex vivo* perfusion of livers with macrosteatosis are anecdotal, especially for NMP (24, 31, 32). The use of de-fatty cocktail during MP preservation for steatotic grafts has been mentioned in the literature but only with animal models (33, 34). This study presents clinical data based on 158 grafts and the risk of causing an untoward event for livers with moderate or severe steatosis. Finally, steatotic livers might develop HCC, even skipping the cirrhotic stage (35, 36), and the risk of HCC recurrence on steatotic graft used in HCC candidates is unknown.

If the results from marginal grafts implanted after MP preservation are undoubtedly promising at short and long term, the definition of “marginal graft” needs to be reformulated in the era of MP. Different criteria and cut-offs have been used to label an ECD but both HMP and NMP seem to have pushed many donor boundaries (17, 37). The list of parameters in Table 5 might be helpful to track this new classification. Indeed if donors have at least two of the listed characteristics (DCD, donor > 65 years, BMI > 30, DRI > 1.9, ALT > 104, AST > 134, bilirubin > 1.2 mg/dl, S-CIT > 8 h, macrosteatosis > 30%), there is a significant risk of ineffectively perfusing the graft into the MP or implanting it unsuccessfully compared to grafts from donors with less than two of the above characteristics (27% vs. 9%, *p* = 0.05). As clarified above, NegE includes MP-perfused and discarded plus grafts perfused, implanted, and lost after the transplant. In the literature, graft loss is usually considered as an event to define marginal donors (10, 38); therefore, the listed factors might be not well applicable in this view but they might help the transplant surgeon to match these specific ECD donors, with such potential outcomes and with low disease severity recipients, like patients with HCC.

The study presented the results of using MP routinely, out of trial, without fixed criteria, and in heterogeneous settings. It showed a national overview of the use of *ex vivo* perfusion of grafts in different centers with their own policy on MP and different availability of this tool.

### Early allograft dysfunction for machine-perfused grafts

EAD is a post-transplant complication that can cost the patient's life. Many donor parameters have been shown to be significant in triggering this potentially catastrophic event, such as donor age, BMI, GGT value, duration of S-CIT, and steatosis (5, 39, 40). None of mentioned factors were found to be significant for EAD in this study (Tables 6,7). This again supports the usefulness of MP, as it might indicate that MP clears off all the potential donor risk factors for EAD and any factors before MP treatment, like S-CIT. For MP grafts, EAD could be caused by factors not considered in this analysis, such as implantation time or duration of anhepatic phase. Interestingly, moderate or severe macrosteatosis is also not significant for EAD, despite the fact that EAD group had twice the rate of macrosteatosis >30% compared with noEAD patients (12% vs. 6%, *p* = 0.34) (Table 7).

EAD had a significant impact on patient and graft survival as shown in Figure 3. Therefore, it urges to explore pretransplant EAD risk factors for machine-perfused grafts to reduce this warning complication.

This study has several limitations. (A) It is a multicenter study; therefore, it includes an heterogeneous graft population. Each center follows its own criteria regarding standard ice storage and MP, which were not considered in this analysis. In some cases, MP was used to manage logistical issues (e.g., multiple donors on the same days), rather than to test marginal grafts, and these data were not traceable in the available database. These grafts might have been stored in the ice box. (B) The analysis included both HMP and NMP and this could cause a bias. In any case, so far there is no solid result that clearly endorses the superiority of one of these tools. (C) Most of the recipients have a low MELD score (11 for noEAD, 10 for EAD) and this could cause a selection bias for EAD analysis. (D) Finally, the samples of the main endpoint are small, so a larger population study is needed.

## Conclusions

In conclusion, today MP is an important device to expand the donor pool in the era of very extended marginal grafts. For the first time in the literature, this study showed that a moderate or severe grade of macrosteatosis could be a factor of graft loss post-transplant, even by perfusing these grafts into an MP, or a reason to discard grafts after MP treatment. Macrosteatosis could temper the widespread efficacy of MP, when the rest of the donor bounders have been successfully pushed. Definitely a new donor scoring system is needed in the era of MP grafts.

Finally, MP has been useful in combating the well-known donor and graft risk factors for EAD. This potentially life-threatening complication is still reported; therefore, it is necessary to explore what other factors may trigger it.

## Data Availability

The original contributions presented in the study are included in the article/Supplementary Material, further inquiries can be directed to the corresponding author.
